# Design and Manufacturing of a High-Sensitivity Cutting Force Sensor Based on AlSiCO Ceramic

**DOI:** 10.3390/mi12010063

**Published:** 2021-01-07

**Authors:** Taobo Gong, You Zhao, Yulong Zhao, Lukang Wang, Yu Yang, Wei Ren

**Affiliations:** 1 State Key Laboratory for Manufacturing System Engineering, Xi’ an Jiaotong University, Xi’an 710049, China; gongtaobo@stu.xjtu.edu.cn (T.G.); zhaoyulong@mail.xjtu.edu.cn (Y.Z.); wanglukang@stu.xjtu.edu.cn (L.W.); yangyuu@stu.xjtu.edu.cn (Y.Y.); rw0192@163.com (W.R.); 2 Science and Technology on Applied Physical Chemistry Laboratory, Shaanxi Applied Physics and Chemistry Research Institute, Xi’an 710061, China

**Keywords:** machining status monitoring, cutting force sensor, high-sensitivity, SiAlCO ceramics, embedded

## Abstract

On-line cutting force measurement is an effective way to monitor processing quality, improve processing accuracy, and protect the tool. In high-speed and ultra-precision machining, status monitoring is particularly necessary to ensure machining accuracy. However, the cutting force is very small in high speed and ultra-precision machining. Therefore, high-sensitivity cutting force sensors are needed. Current commercial cutting force sensors have defects such as large volume, low compatibility, and high price. In particular, the sensitivity of cutting force sensor needs to be improved for high-speed and ultra-precision machining status monitoring. This paper provides a possible solution by embedding the sensor in the tool and selecting sensitive materials with high piezoresistive coefficient. In this paper, the structural design of the sensor and the fabrication of the sensitive material SiAlCO ceramic are carried out, and then the sensor is packaged and tested. The test results show that the cutting force sensor’s sensitivity was as high as 219.38 mV/N, which is a feasible way to improve cutting force sensor’s compatibility and sensitivity.

## 1. Introduction

Intelligent manufacturing requires high-end CNC (Computer Numerical Control) machine tools, while the automatic and adaptive processing of high-end CNC machine tools requires cutting state monitoring. Cutting status monitoring is essential for high-speed and ultra-precision machining. Commonly used cutting condition monitoring technologies mainly include: cutting force measurement technology [1], cutting temperature measurement technology [2], cutting vibration measurement technology [3], etc. Cutting force is a physical phenomenon produced by the direct contact between the tool and the workpiece during the cutting process. Therefore, cutting force measurement is a direct and effective way to monitor cutting conditions [4]. The cutting force is closely related to cutting quality [5], efficiency [6], and tool wear [7]. By measuring the cutting force, real-time monitoring of the cutting status can be realized, so that the cutting parameters can be adjusted to achieve the purpose of improving cutting quality, increasing cutting efficiency, and increasing tool life. Cutting force measurement technology is currently the most widely researched and applied cutting condition monitoring technology [8,9].

Cutting force measurement methods can be divided into tool holder-based methods, table-based methods, and tool-based methods. Xie, Z. et al. use capacitance sensors and accelerometers to make a smart tool holder for cutting process monitoring. The maximum sensitivity of the smart tool holder is 2353 aF/N [10]. The actual measurement of milling force is carried out using the smart tool holder, and the effect is relatively good. However, the entire measurement structure is relatively large, and the compatibility needs to be improved. Liu, M. et al. develop a tool holder based on fiber Bragg grating sensor for cutting force measurement, with a maximum sensitivity of 1.08 με/N and a natural frequency of 320 Hz [11]. The tool holder was designed as an octagonal ring structure, and the sensor was pasted on the sensitive structure of the tool holder. Husaini et al. attached a strain gauge to the octagonal ring and used four octagonal rings to form a tool holder to measure the cutting force, which can measure the three-axis cutting force. The measuring range of the dynamometer was 2.9 kN, the natural frequency was 766 Hz, and the sensitivity was 31.3 × 10^−3^–172.4 × 10^−3^ mV/N [12]. When the dynamometer is used to measure the actual cutting force, it can be installed on an ordinary lathe, but it is troublesome to install in a CNC machine, and the sensitivity of the dynamometer is relatively small. Luo, M. et al. embedded a PVDF thin-film sensor in the workbench to measure the milling force. The PVDF thin-film sensor had a sensitivity of 43.94 pC/N, and its operating frequency range was 0.8 Hz to 10 kHz [13]. The measurement result of this sensor was basically consistent with the commercial sensor. But the sensor needs to be equipped with an expensive charge amplifier for measurement. Uddin, M.S. et al. attached the strains to the octagonal ring and the four-corner ring, respectively, and then made worktables to measure the cutting force. Through experimental comparison, the accuracy of the force gauge based on the square ring was higher [14]. Both holder-based and table-based measurement methods were indirect measurements of cutting force. In order to directly measure the cutting force, many scholars conducted research on tool-based measurement methods. Huang, J. et al. developed a smart tool based on fiber Bragg grating sensor for cutting force measurement. Pasting sensors on the four sides of the tool, the maximum sensitivity was 3.66 N/pm, and the maximum frequency was 6383.2 Hz [15]. However, the sensor occupied a lot of positions on the tool holder, which made the installation of the tool inconvenient, and the sensitivity of the sensor needs to be further improved. Li, X. et al. slotted the middle position of the tool handle, and then embedded the nickel-chromium film sensor in the slot. The piezoresistive effect of NiCr film was used to measure the cutting force. The sensitivity of the sensor was 0.00518 mV/N, which was relatively small [16]. Although tool-based measurement methods are straightforward, the current research on tool-based cutting force sensors showed relatively low sensitivity, so there is an urgent need to study tool-based high-sensitivity cutting force sensors. Therefore, this paper plans to develop a small and embedded cutting force sensor, which is embedded in the tool for measuring cutting force. The sensitive material of the sensor adopted SiAlCO ceramic material with high resistance coefficient. The sensor was embedded in the tool instead of the tool pad to achieve high sensitivity of the sensor.

SiAlCO ceramic is a kind of polymer-derived ceramic (PDC). Polymer precursor ceramics are a new type of ceramic material obtained directly by pyrolyzing polymer precursors. Because its internal structure is mainly composed of an amorphous matrix phase and a free carbon phase, PDC ceramics have many unique and excellent properties including excellent high-temperature thermal stability [17], high-temperature semiconductor characteristics [18], high creep resistance, and huge piezoresistive characteristics [19]. Especially the gauge factor of SiAlCO ceramic is as high as 7000–16,000. These outstanding properties have made PDCs extensively studied in many aspects, and their applications mainly include microelectromechanical systems (MEMS) [20], high-temperature sensors [21,22], pressure sensors [23], etc. Many scholars have used the piezoresistive effect of PDC ceramics to make pressure sensors. Cheng, H. et al. developed a wireless passive pressure sensor using SiAlCN ceramics. The sensor can measure the pressure of 0–5 N at a high-temperature of 800 °C [24]. Leo, A. et al. fabricated the SiCN ceramic film and used it to make a pressure sensor [23]. Li, N. et al. used SiAlCO ceramic as the sensitive material to make a pressure sensor and conducted test experiments. Experiments show that the sensor had high accuracy, stability, and repeatability, and the SiAlCO ceramic had the potential for high-temperature applications. The sensitivity of the pressure sensor was 58.3 mV/MPa [25]. SiAlCO ceramic is an excellent choice for sensitive materials for cutting force sensors.

## 2. Materials and Methods

### 2.1. Principle of Sensor Measurement

The sensor developed in this paper was based on the piezoresistive effect. When a cutting force is applied to the sensor, it will cause the sensor’s sensitive resistance to change, and the sensor measuring circuit will output a corresponding voltage change. The change of sensor resistance is related to the piezoresistivity of SiAlCO ceramics. For a material with a length of *L*, a cross-section of *S*, and resistivity of *ρ*, the relationship between the change in resistance and the force *F* is:

(1) $$\frac{dR}{R}=\pi \sigma +\frac{dL}{L}+2\mu \frac{dL}{L}=(1+2\mu +\pi E)\varepsilon =GF\varepsilon$$

where *π* is the piezoresistive coefficient of the material, *μ* is the Poisson’s ratio, *ε* is the strain, *σ* is the stress, *E* is the elastic modulus, and *GF* is the gauge factor. The gauge factor *GF* of SiAlCO ceramic material is as high as 7000–16,000, so that when SiAlCO ceramic material is under force *F*, the change in resistance will be noticeable.

**Figure 1:**
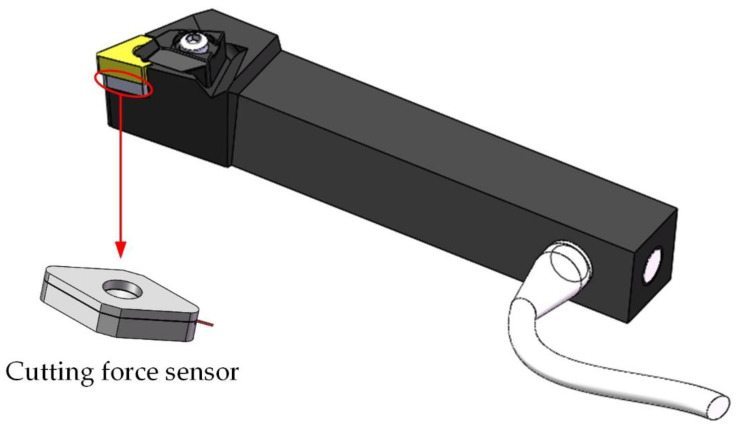
The overall structure of the cutting force measurement.

The overall structure of the embedded high-sensitivity cutting force measurement device researched in this paper included the cutting force sensor and the smart tool, as shown in Figure 1. The cutting force sensor was inserted into the smart tool instead of the tool pad. Then the tool was capable of simultaneously cutting and measuring cutting forces without changing the shape of the tool, which makes the tool have good compatibility and versatility with the machine tool system.

**Figure 2:**
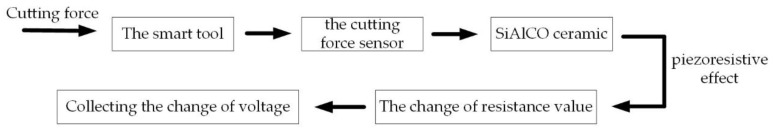
The working principle of the cutting force measurement.

The working principle of cutting force measurement is shown in Figure 2. During the cutting process, the cutting force *F* is applied to the tool and then transmitted to the cutting force sensor. Because the sensitive material SiAlCO ceramic of the cutting force sensor has the piezoresistive effect, the resistance value of the SiAlCO ceramic will change with the cutting force *F*. Then, the voltage of SiAlCO ceramics will change. The change of voltage is collected by the data acquisition card. Then, after the signal is transmitted and processed, the cutting force *F* will be displayed and stored on the computer.

### 2.2. Structure Design

**Figure 3:**
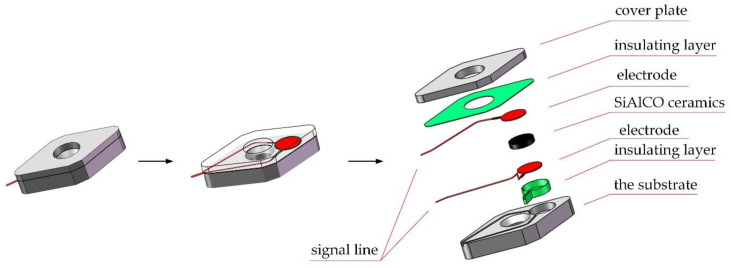
The structure of the cutting force.

**Figure 4:**
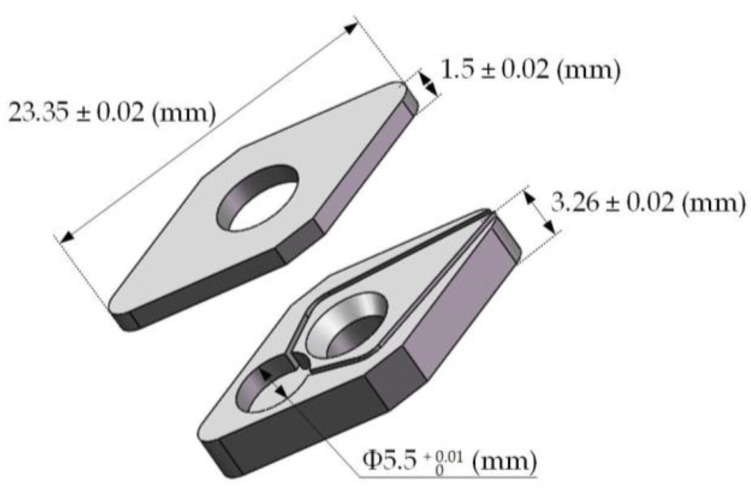
Dimensions of the substrate and cover plate.

As shown in Figure 3, the cutting force sensor developed in this paper mainly included a substrate, a cover plate, and a sensitive unit. The sensitive unit mainly included: a sensitive resistor, two electrodes, signal lines, and insulating layers. The structural design mainly was divided into three parts: the substrate and cover plate, sensitive unit, and the smart tool. The material selected for the substrate and the cover plate was 304 stainless steel. The mechanical properties of 304 stainless steel were very similar to those of the original shim material, and it was easier to process and cheaper. The specific dimensions of the substrate are shown in the Figure 4, and the external dimensions were the same as the original tool pad. The sum of the thickness of the substrate and the cover plate was the same as that of the original tool pad. The sensitive unit of the sensor adopts a sandwich structure and consisted of two electrodes and a SiAlCO ceramic wafer in the middle. The electrodes and the ceramic wafer were a round shape of Φ5.5 mm. The electrodes and the ceramic wafer were bonded with conductive silver paste.

**Figure 5:**
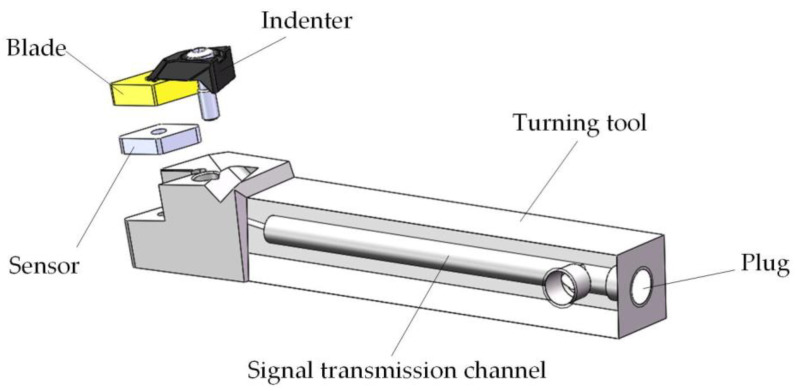
The structure of the smart tool.

The structural design of the smart tool mainly considered the transmission mode of the sensor signal line. Inserting the sensor into the tool instead of the tool pad and adopting the wired transmission method had the advantages of strong anti-interference ability and good stability. Moreover, since the sensor replaced the tool pad, it was very close to the source of cutting force. The sensor was easily impacted by cutting heat, cutting fluid, and chips. Therefore, the signal transmission data line of the cutting force sensor could not be exposed, and it needed to go through the inside of the smart tool and connect to the data acquisition card. This required the design of signal transmission channels inside the smart tool. The smart tool structure specifically included: cutting force sensor, blade, indenter, signal transmission channel, and plug, as shown in the Figure 5.

**Figure 6:**
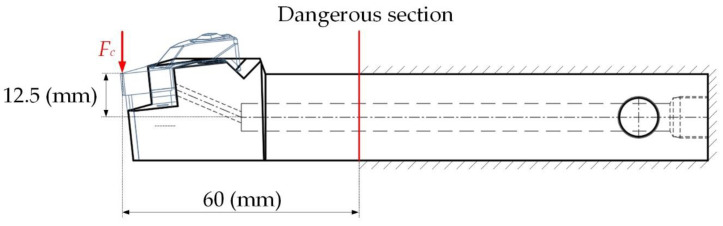
Cutting force acting on the tool.

In order to check whether the cutting tool embedded with the cutting force sensor can work safely in actual cutting, it was necessary to conduct a safety check on the tool. According to the actual cutting condition of the tool, the situation when the tool is under force was established, as shown in the Figure 6. Under the action of the main cutting force Fc, the dangerous section of the tool was as shown in the Figure 6. The dangerous section of the tool was at the end of the gripper. The horizontal distance between the force point and the dangerous section was 60 mm; the distance between the force point and the neutral axis of the section was 12.5 mm. The tool material was 42CrMo alloy structural steel. The bending stress and shear stress at any point in the cantilever beam structure can be obtained by Equations (2) and (3). The third strength theory [26] was selected for strength check, and the check formula is shown in Equation (4).

(2) $$\sigma =\frac{M\;\times \;y}{I_{Z}}$$

(3) $$\tau =\frac{{Q\;\times \;S}_{z}^{*}}{{bI}_{z}}$$

(4) $$\sigma_{r}=\sqrt{\sigma^{2}+{4\tau }^{2}}\leq \frac{\sigma_{s}}{n}=\left[\sigma \right]$$

where *M* is the bending moment, *y* is the distance from a certain point to the neutral axis, *I_z_* is the moment of inertia of the axis on the cross-section, *Q* is the shear force on the cross-section where the point is located, and S_z_* is the static cross-section relative to the neutral axis, *b* is the section width, *σ_r_* is the equivalent stress, *σ_s_* is the yield strength of the material, [*σ*] is the allowable stress, and *n* is the safety factor.

Calculated from the above formula, the maximum equivalent normal stress σ of the dangerous section was 4.63 × 10^4^ *F_c_*, and the maximum equivalent shear stress wa *τ* = 4.34 × 10^3^ *F_c_*. The measuring range *F_c_* of the cutting force sensor designed in this paper was 200 N. According to Equation (4), the equivalent stress *σ_r_* of the dangerous section of the tool structure under the full scale was calculated as 9.42 MPa. The yield strength *σ_s_* of the tool material 42CrMo was 920 MPa [27], and the safety factor *n* was set to 2, then the allowable stress [*σ*] 460 MPa, the equivalent stress *σ_r_* of the dangerous section of the tool structure was much smaller than the allowable stress [*σ*]. Therefore, the tool structure designed in this paper was safe and reliable under the load of the full range of the cutting force sensor.

### 2.3. Sensitive Unit Manufacturing

**Figure 7:**
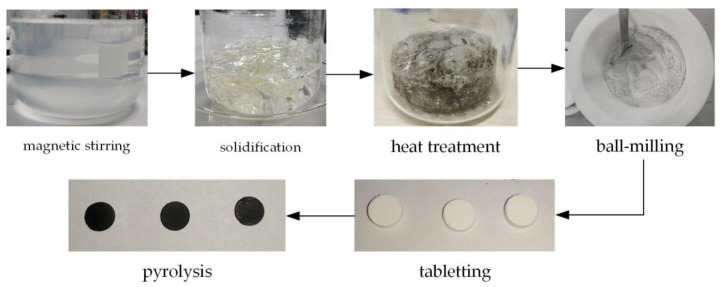
SiAlCO ceramic production process.

**Figure 8:**
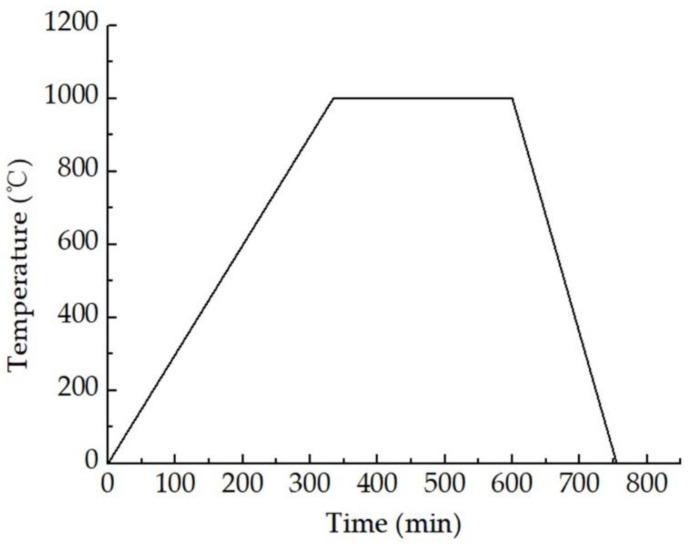
SiAlCO ceramic pyrolysis temperature curve.

The production of SiAlCO ceramics used aluminum sec-butoxide and silicone resin as raw materials and was synthesized through pyrolysis. The silicone resin used belongs to a high-carbon polysiloxane containing phenyl groups, and aluminum sec-butoxide was the source of aluminum. The production steps of SiAlCO ceramics is shown in Figure 7, and the details are as follows: (1) mixing the silicone resin with ethanol at a mass ratio of 2:1, then adding 5% by mass of ATB; (2) stirring the mixture at room temperature at 800 rpm for 6 h to obtain SiAlCO precursor solution; (3) solidifying the SiAlCO precursor solution at 150 °C and heat-treating the solids in ultra-high purity argon at 350 °C for 4 h; (4) ball-milling the solids to obtain a granular powder less than 1 μm; (5) mixing the powder with the SiAlCO precursor solution in a volume ratio of 40:1 and compressing the powder mixture into a Φ5.5-mm wafer, then compacting the wafer by a cold isostatic press; (6) pyrolyzing at 1100 °C under ultra-high purity argon gas following the pyrolysis temperature curve shown in Figure 8 to obtain the SiAlCO ceramics [28].

**Figure 9:**
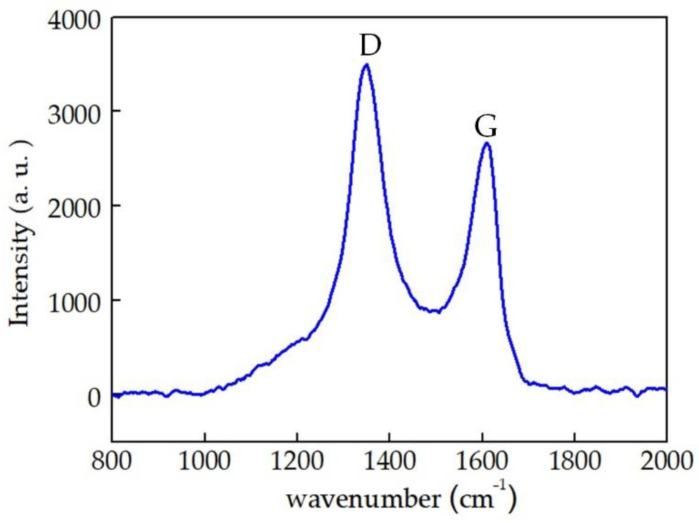
Raman spectra of the SiAlCO ceramic sample.

Free carbon is an important factor in piezoresistive effect of SiAlCO ceramics. After obtaining the SiAlCO ceramic samples, we analyzed whether the samples contained free carbon or not. The test was carried out by Raman spectrometer. As shown in the Figure 9, the Raman spectrum contains D peak and G peak, which are characteristic peaks of carbon atoms. SiAlCO ceramics had obvious characteristic peaks around 1350 cm^−1^ and 1600 cm^−1^, corresponding to peak D and peak G, respectively. The appearance of peak D was caused by the breathing vibration mode of sp^2^ hybridized ring carbon atoms. Peak G was caused by the stretching vibration mode of sp^2^ hybrid carbon atoms in the plane [29]. All samples had D peak and G peak, which proved the existence of free carbon in the samples. 

### 2.4. Sensor Packaging

After the SiAlCO ceramic was manufactured, the next step is to package the cutting force sensor so that the sensor can be used for actual cutting force measurement. The actual application environment of the cutting force sensor was more complicated, including the corrosion of the cutting fluid, the vibration of the machine tool system, the impact of high-temperature chips, and the processing noise, etc. These interferences will adversely affect the dynamic and static performance, reliability, and long-term stability of the cutting force sensor in actual use. These adverse effects need to be reduced or eliminated through the packaging of the cutting force sensor. According to the complex actual application environment of the cutting force sensor, the packaging principles of the sensor were designed as follows: (1) after packaging, the sensor should realize the connection of the sensitive resistor and the measuring circuit and the corrosion resistance, high-temperature resistance and insulation protection of the sensitive resistor; (2) the cover plate is rigidly connected with the substrate into one body, so that the packaged sensor and the tool pad are consistent in overall shape and size and isolate the sensor from the outside.

**Figure 10:**
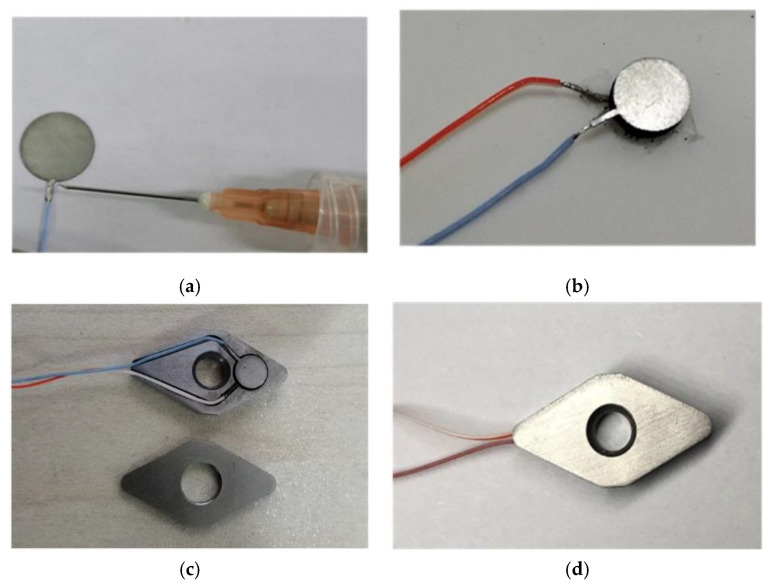
Process of the cutting force sensor packaging: (**a**) connecting the wires and electrodes; (**b**) contacting the electrodes with the SiAlCO ceramic; (**c**) spraying the insulating paint on the surface of the cover plate and the substrate; (**d**) the SiAlCO ceramic with the electrodes are put into the groove of the substrate, and the cover plate is welded to the substrate using laser welding.

The packaging steps of the sensor are shown in Figure 10: (a) The wires and electrodes are connected by conductive silver paste; (b) the electrodes are in close contact with the surface of the SiAlCO ceramic through the silver paste; (c) the insulating paint is sprayed on the surface of the cover plate and the substrate as an insulating layer; (d) the SiAlCO ceramic with the electrodes are put into the groove of the substrate, and then the cover plate is welded to the substrate using laser welding. The cutting force sensor packaged is shown in Figure 10d. In the following research, the performance of the sensor will be tested.

### 2.5. Sensor Test Plan

**Figure 11:**
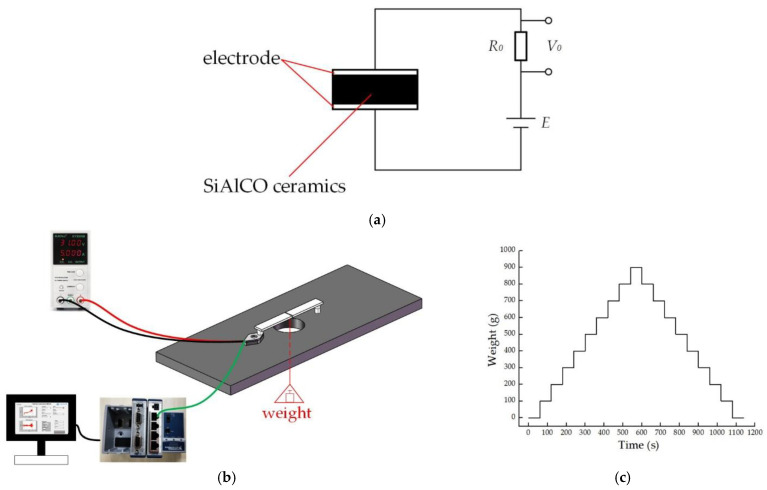
(**a**) The circuit schematic diagram of the test; (**b**) diagram of loading weights; (**c**) weight loading curve.

Figure 11a is the schematic diagram of the circuit set up for the experiment. The sensor was connected to the outside circuit by two signal lines. The two signal lines were connected with the fixed value resistor *R*_0_ and the power supply *E*. As shown in Figure 10b, the force *F* was generated by loading weights. When force *F* was applied to the sensor, it transferred to the SiAlCO ceramic. Due to the piezoresistive effect, the resistance value of SiAlCO ceramics changed, resulting in a change in the voltage value of the fixed value resistor *R*_0_. The performance of the sensor can be obtained by measuring the voltage value of the fixed value resistor *R*_0_. The data acquisition system was used to collect the voltage of the fixed value resistance.

As shown in Figure 11b, the performance test of the sensor was carried out on the self-built force sensor calibration platform. The testing device mainly included the calibration platform, weights, the weight plate, the beam, power supply, the data acquisition system, and a computer. The power supply voltage was 5 V. The specific test steps were as follows: (1) connecting the circuit according to the circuit schematic shown in Figure 11a; (2) placing the beam according to Figure 11b, with one end of the beam on the sensor; (3) adjusting the weight plate position so that it is in the middle of the beam; (4) turning on the power supply and adding the weights to the weight plate one by one. The weight increased from 0 to 900 g, each time increasing by 100 g. Each weight was maintained for 60 s, as shown in the Figure 11c. At the same time, the data acquisition system collected the voltage output by the sensor; (5) removing the weights from the weight plate one by one. The weight of the weight was reduced from 900 to 0 g, each time by 100 g; (6) repeating the steps (4) and (5) several times.

### 2.6. The Data Acquisition System

The data acquisition (DAQ) system consists of a data acquisition card, a chassis, and an upper computer acquisition program based on LabVIEW 2014 (NI, Austin, TX, USA). The data acquisition card used the NI 9218 data acquisition module of National Instruments, and the main parameters of it are shown in the Table 1. After the input signal of the channel of the collection card was buffered and conditioned, it was sampled by the analog-to-digital converter (ADC). The NI 9218 data acquisition module was used with the NI CompactDAQ 9174 chassis (NI, Austin, TX, USA), which can be directly connected to the computer via USB. This paper details a data acquisition program based on LabVIEW on the host computer. The functions of the data acquisition program included the setting of sampling parameters, the real-time acquisition of voltage data, the real-time display of data, and the storage of data. The sampling mode set in this paper was continuous sampling and the sampling frequency was 12.8 kHz.

## 3. Results and Discussion

**Figure 12:**
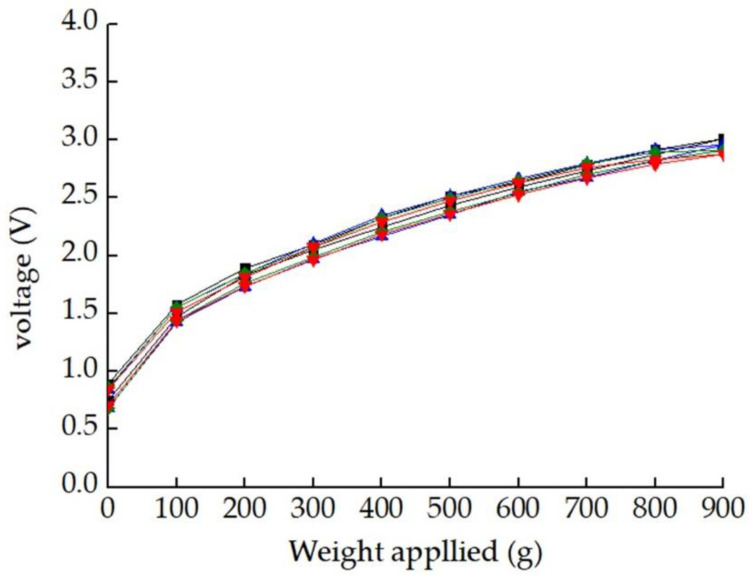
Relationship between the output voltage and the weight applied.

Through the static calibration experiment, the input and output characteristic curves of the sensor could be obtained, as shown in Figure 12. The sensor could produce almost identical responses in all of the four cycles and showed a very good repeatable performance, and the repeatability *ξ_R_* was calculated by Equation (5) [30] as 12.71%. It can be seen from the figure that the sensor showed good linearity within the calibration range of 100–900 g, and the linearity error *ξ_L_* of the sensor within this range was calculated to be 10.10% obtained by Equation (6). However, the linearity in the range of 0–900 g was not ideal, which was 22.70%. This problem may be caused by poor contact between the SiAlCO ceramic and the electrode and the package cover. In order to solve this problem, in future research, the pre-tightening force will be added in the sensor packaging process. In addition, according to the input and output characteristic curves of the sensor, its sensitivity *s* was calculated to be 219.38 mV/N, obtained by Equation (7). According to Table 2, the sensitivity of the cutting force sensor based on SiAlCO ceramic was significantly higher than that of other sensors.

(5) $$\xi_{R}=\frac{\lambda S\;}{Y_{FS}}\times 100\%$$

(6) $$\xi_{L}=\frac{\left|Y_{i}-Y_{i}^{\prime }\right|}{Y_{FS}}\times 100\%$$

(7) $$s\;=\frac{\;∆f}{∆p}$$

where *λ* is the inclusion factor, the value is 95%, *S* is the standard deviation of the sub-sample in the entire measurement range, *Y_i_* is the average value of one point, *Y_i_^′^* is the value of one point, *Y_FS_* is full-scale output, ∆*f* is The increment of output, ∆*p* is the increment of input.

**Figure 13:**
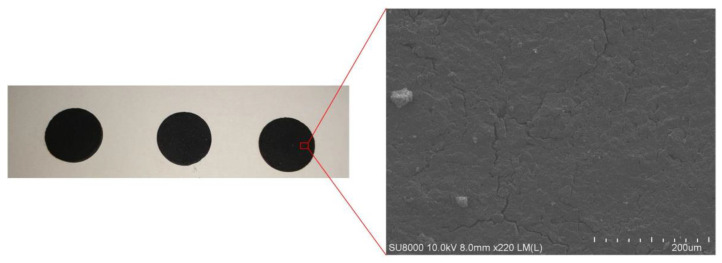
SEM picture of SiAlCO ceramics.

**Figure 14:**
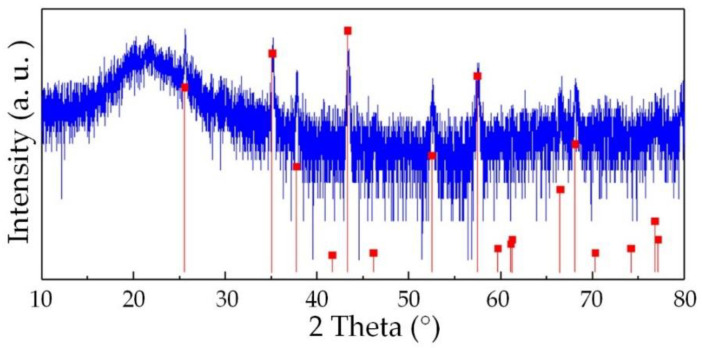
XRD pattern of SiAlCO ceramics.

In order to research the reason for the poor repeatability of the sensor, the SiAlCO ceramic samples were analyzed by a scanning electron microscope (SEM, SU8010, Hitachi, Japan) and x-ray diffractometer (XRD, D8 ADVANCE A25, Bruker, Germany). SEM was used to observe the surface morphology of the SiAlCO ceramic sample. As shown in the Figure 13, small gullies and holes on the surface of SiAlCO ceramics were observed in the SEM picture. It can be known that the SiAlCO ceramic samples produced had low density. This led to poor sensor repeatability. Figure 14 is the XRD pattern of the SiAlCO ceramic sample. The red lines in the spectrum are the possible diffraction peaks of Al_2_O_3_ crystals [33]. From the XRD pattern, eight peaks of Al_2_O_3_ crystals coincided with the diffraction peaks that appear in the X-ray diffractometer spectrum of the SiAlCO ceramic measured. It indicates that Al_2_O_3_ crystals appeared in the prepared SiAlCO ceramics. The Al_2_O_3_ crystal will affect the conductivity of the sample, resulting in poor sensor repeatability. Therefore, the quality of SiAlCO ceramics will be improved from two aspects in subsequent research. The first measure is to improve the quality of the tabletting, including the use of smaller particle size powder and higher pressure. The second measure is to improve the process to prevent the sample from contacting oxygen during the heat treatment process.

## 4. Summary and Conclusions

Monitoring high-speed and ultra-precision machining processes requires cutting force sensors with high sensitivity. Currently available cutting force sensors have the problems of insufficient sensitivity and low compatibility with machine tool systems. In response to these two problems, this paper designs a high-sensitivity cutting force sensor using SiAlCO ceramic as the sensitive material, and embeds the sensor into the tool to improve compatibility with the machine tool. This paper presents SiAlCO ceramic samples. The SiAlCO-based cutting force sensor was manufactured, packaged, and tested. The following conclusions can be drawn:

(1) The cutting force sensor developed in this paper has easy-to-obtain manufacturing materials, mature processing methods, and mature packaging technology. SiAlCO ceramic has the potential to be a sensitive element of a high-sensitivity cutting force sensor. According to the test results, the sensitivity of the cutting force sensor based on SiAlCO ceramic reached 219.38 mV/N.
(2) Preliminary research results showed that the linearity and repeatability of the cutting force sensor are not ideal, which was related to the purity and internal defects of the AlSiCO sample.
(3) The next step is to improve and adjust the preparation process to solve the problems of purity and internal defects of AlSiCO samples to ensure the linearity and repeatability of the sensor. At the same time, in order to improve the performance of the sensor, a pre-tightening force will be added during the packaging process to make the working area of the sensor avoid areas with poor performance, and the cutting force sensor will be used in the actual cutting experiment to study the effects of the process parameters on the measured force components.

## Figures and Tables

**Table 1:** The main parameters of the DAQ system ^1^.

The Data Acquisition Card	Number of Channels	ADC Resolution	Sampling Mode	Frequency
NI 9218	2 analog input channels	24 bits	Simultaneous	13.1072 MHz

^1^ More detailed parameters refer to the website: www.ni.com.

**Table 2:** Sensitivity comparison of different cutting force sensors.

Sensor	Sensitivity
This work	219.38 mV/N
[12] Husaini	31.3 × 10^−3^–172.4 × 10^−3^ mV/N
[16] Li, X.	5.18 × 10^−3^ mV/N
[31] Zhao, Y.	0.31 mV/N
[32] Panzera, T.H.	1.32 × 10^−3^ mV/N
